# Screening of Lactic Acid Bacteria Isolated from Fermented Cowpea and Optimization of Biomass Production Conditions

**DOI:** 10.3390/foods14020150

**Published:** 2025-01-07

**Authors:** Hong Xu, Danyang Li, Xue Jiang, Qi Pei, Zhengqin Li, Philippe Madjirebaye, Mingyong Xie, Tao Xiong, Zhanggen Liu

**Affiliations:** 1 State Key Laboratory of Food Science and Resources, No. 235 Nanjing East Road, Nanchang 330047, China; 18397836518@163.com (H.X.); l12250166@163.com (D.L.); olivia001216@163.com (X.J.); 15798036686@163.com (Q.P.); lzq012500@163.com (Z.L.); madjirebaye22@gmail.com (P.M.); myxie@ncu.edu.cn (M.X.); xiongtao0907@163.com (T.X.); 2 School of Food Science & Technology, Nanchang University, No. 235 Nanjing East Road, Nanchang 330047, China; 3 International Institute of Food Innovation International, Nanchang University, Luozhu Road, Xiaolan Economic and Technological Development Zone, Nanchang 330200, China; 4 International Institute of Food Innovation Co., Ltd., Nanchang University, Luozhu Road, Xiaolan Economic and Technological Development Zone, Nanchang 330200, China

**Keywords:** *Pediococcus pentosaceus*, screening lactic acid bacteria, performance optimization, biomass production

## Abstract

Considering the four characteristics of strains, including acid production, acid tolerance, salt tolerance, and nitrite degradation rate, *Pediococcus pentosaceus* NCU006063 was selected as the fermentation agent, and the medium composition of *Pediococcus pentosaceus* NCU006063 was optimized using Plackett–Burman and central composite rotational design. Three of the seven factors studied in the Plackett–Burman design significantly affected the viable counts. A central composite rotational design was used to optimize the significant factors and generate response surface plots. Using these response surface plots and point predictions, the optimal factors were soy peptone (38.75 g/L), FeSO_4_ (0.10 g/L), and VB_7_ (20 g/L). In addition, the optimized incubation conditions were a temperature of 39 °C, an initial pH value of 7, and an inoculation volume of 3%. The optimized biomass production parameters were a constant pH (6.5), neutralizing agent types (25% NH_3_·H_2_O), and gas types (N_2_). Under these optimal conditions, *Pediococcus pentosaceus* NCU006063 exhibited a great viable bacterial count of up to 2.65 × 10^10^ CFU/mL, which is 9.71 times higher than that of MRS broth (2.73 × 10^9^ CFU/mL). These results demonstrated that the *Pediococcus pentosaceus* NCU006063 strain has excellent potential as a fermentation agent and can provide a theoretical base for the in-depth exploration and promotion of fermented cowpea use in human diets.

## 1. Introduction

Factory-fermented cowpeas are cowpeas freshly soaked in a brine with a salt content of 7–9%, compressed with wooden sticks, and fermented in a relatively open environment. Because of its crisp, salty, sour, and delicious taste, as well as its abundance of amino acids, vitamin C (VC), phenolic compounds, and carbohydrates [1,2,3], fermented cowpeas are well liked by consumers. With the increasing demand for fermented cowpeas, the demand for cowpea starters is also increasing. However, the fermentation process is susceptible to the growth of stray bacteria [4], and the high salt process also carries risks like nitrite accumulation [5,6]. Therefore, it is challenging to ensure the flavor quality of fermented cowpeas.

In order to improve the quality and production safety of cowpeas and facilitate industrialization and large-scale production, it is necessary to inoculate lactic acid bacteria to ferment cowpeas in the future. An excellent starter is a crucial assurer of a superior quality in fermented foods, and obtaining high-density bacteria is a key step in starter preparation [7]. A key indicator of cowpea maturity is how well the strain produces acid throughout the fermentation process. Lactic acid bacteria face difficulties growing and reproducing because of the high acid and high salt environment present during the fermentation of cowpeas. Additionally, the high salt environment is harmful due to nitrite buildup. Nitrite can be broken down by acid degradation [8] or enzymatic hydrolysis [9] in lactic acid bacteria. Therefore, the special strains of lactic acid bacteria were selected in this experiment based on their ability to produce acid, acid tolerance, salt tolerance, and nitrite degradation.

A variety of bacteria were involved in the vegetable fermentation process, but generally the main probiotic bacteria were lactic acid bacteria, which are inhibited by spoilage bacteria [10]. During fermentation of sour asparagus, lactic acid bacteria and *Lactococcus* dominate the whole microbial community [11]. Probiotics played an important role in the fermentation process of foods, producing flavor components and ensuring the quality and safety of fermented foods [12]. Traditional culture methods had a series of problems, such as low bacterial concentration, differences in quality, and high production costs [13]. Therefore, the biomass production of lactic acid bacteria is necessary. Biomass production refers to the culture method that uses specific culture technology and equipment or changes the culture conditions or adds some reagents to increase the cell density more than 10 times compared with the traditional culture method [14,15,16]. The ordinary culture method can increase cell density, lower strain culture costs, and shorten production time [13]. Presently, optimizing the components of the culture medium (carbon source, nitrogen source, inorganic salt, and growth factor, etc.) and the culture conditions (culture temperature, pH value of fermentation broth, inoculum amount, etc.) are the main focus of biomass production [17].

To produce direct-injection fermentation agents, this study screened lactic acid bacteria with excellent fermentation performance, optimized the composition of the culture medium for *P. pentosaceus* NCU006063, and explored the optimal culture conditions with high bacterial concentration under high-density, culture temperature, pH, and other conditions.

## 2. Materials and Methods

### 2.1. Samples Collection

Twelve samples of fermented cowpea were obtained from a natural fermentation factory in Yunnan Province. After collection, the fermented cowpea samples were placed into sterile plastic containers, transferred then into 50 mL sterile centrifuge tubes, and maintained at 4 °C.

### 2.2. Isolation and Screening of LAB Strains from Fermented Cowpea

The isolation of LAB from fermented cowpea samples was performed using the method of Chang-Kyung Oh et al. [18]. A quantity of 100 μL of fermented cowpea brine was taken in 900 μL of sterilized saline, shaken well, serially diluted to a suitable gradient, and evenly spread on MRS AGAR plates containing bromocresol purple. After incubation at 37 °C for 48 h, a single colony (yellow color around the colonies) was selected by Qpix 420 (Molecular Devices, UK Limited, Wokingham, UK) for isolation and purification for further screening experiments.

### 2.3. DNA Extraction and PCR Amplification for 16S rRNA Gene Sequence

Microbial genomic DNA was extracted from fermented cowpea samples using a rapid genomic DNA extraction kit (Sangon Biotech, Shanghai, China) according to the manufacturer’s instructions, and 16S rRNA gene sequence PCR amplification was performed using the forward primer 27F (5′-AGA GTT TGA TCC TGG CTC AG-3′) and reverse primer 1512R (5′-CGG CTA CCT TGT TAC GAC T-3′) [19]. The PCR amplification conditions used are as follows: initial denaturation at 94 °C for 4 min, 35 cycles of denaturation at 94 °C for 30 s, annealing at 55 °C for 30 s and extension at 72 °C for 40 s, and a final extension at 72 °C for 10 min. The PCR products were sent to Sangon Company (Shanghai, China), and the sequences were analyzed using the BLAST algorithm.

### 2.4. Preliminary Selection of Strains

#### 2.4.1. Determination of Acid Production Capacity

The activated LAB isolates were inoculated into MRS broth (inoculation volume 2%) and cultured at 37 °C. The pH value and TA of the MRS were measured by using a digital pH meter (PHS-25; Shanghai Precision Scientific Instruments Company, Shanghai, China) for 0, 2, 4, 6, 8, 10, 12, 24, 36, and 48 h. Titratable acidity (TA) values, expressed as grams of lactate per 100 mL saline, were measured by titrating 0.1 N NaOH to pH 8.2 ± 0.2 [20,21]. The acid production rate can be calculated using the following equation:

$$\mathrm{Titratable}\;\mathrm{acidity}\;(g/\mathrm{kg})\;=\frac{C\times V\times 0.09}{V_{0}}\times 1000$$

where C: concentration of NaOH (moL/L); V: volume of NaOH solution consumed by titration (mL); V_0_: volume of the sample (mL); 0.09: total acid conversion coefficient.

#### 2.4.2. Determination of Acid Tolerance Capacity

Based on the results of acid production capacity, further testing will be conducted on the acid resistance, salt resistance, and nitrite degradation ability of lactic acid bacteria strains. As previously described, LAB resistance at low pH was examined [22]. Briefly, LAB was cultured in MRS broth for 24 h at 37 °C, and their pureed bacteria were collected by centrifugation (10,000× *g*, 5 min, 4 °C), washed twice with PBS buffer (pH 7.2), and resuspended in PBS solution, and then adjusted to pH 2.0 and pH 3.0. Viable counts of LAB after 0 and 3 h incubation were determined by serial dilutions using the standard plate method on MRS AGAR (Biomaxima, Lublin, Poland) for incubation at 37 °C for 48 h. The results are expressed as the mean of three replicates and are expressed as the logarithm of the number of microorganisms. The survival rate is calculated using the following formula:

$$\mathrm{Survival}\;\mathrm{rate}\;(\%)\;=\frac{\mathrm{Viable}\;\mathrm{count}\;(3\;h)}{\mathrm{Viable}\;\mathrm{count}\;\mathrm{of}\;\mathrm{countrol}\;\mathrm{group}\;(0\;h)}\times 100\%$$

#### 2.4.3. Determination of Salt Tolerance Capacity

LAB isolates grown in MRS broth containing 0 and 10% NaCl were inoculated (2%) in 10 mL of fresh MRS for 24 h at 37 °C. Subsequently, the number of viable counts was calculated to analyze the salt tolerance of LAB. The results were expressed as the mean of three replicates and are expressed as the logarithm of the number of microorganisms.

#### 2.4.4. Determination of Degrade Nitrite Capacity

The ability of LAB to degrade nitrite in MRS broth was determined by the method of Dodds-Collins-Thompson [23]. Nitrite was added to the test tube with 10 mL MRS broth medium to the final concentration of 120 mg/mL and the tubes with 200 µL of a 24 h culture of each strain. After incubation at 37 °C for 24 h, 5 mL of bacteria culture was homogenized with 12.5 mL saturated borax solution. The mixture was heated at 100 °C for 15 min, followed by adding 5 mL 10^6^ g/L potassium ferrocyanide solution and 5 mL 220 g/L zinc acetate solution. After the supernatant was shaken to precipitate the protein, 2 mL 4 g/L of sulphonic acid solution and 1 mL 2 g/L of N-(1-naphthol) ethylenediamine dihydrochloride were added. After 15 min, the absorbance was measured by spectrophotometer at 538 nm wavelength. Standard curves were prepared using a series of standard nitrite solutions. The nitrite concentration was calculated from the standard curve and expressed as mg/kg. All the vessels used for incubation were sterilized by autoclaving at 121 °C for 15 min. The nitrite degradation rate was calculated using the following equation:

$$\mathrm{Nitrite}\;\mathrm{degradation}\;\mathrm{rate}\;(\%)\;=\frac{\mathrm{nitrite}\;\mathrm{concentration}\;(24\;h)}{\mathrm{nitrite}\;\mathrm{concentration}\;(0\;h)}\times 100\%$$

#### 2.4.5. Principal Component Analysis for the Screening of Bacterial Strain

Principal component analysis was applied to objectively evaluate strains from multiple indicators, including acid production capacity, acid tolerance capacity, salt tolerance capacity, and degraded nitrite capacity. The candidate LAB strains were selected based on the comprehensive scores generated by PCA. The PCA was performed by SPSS 27.0 (Analytical Software, Armonk, NY, USA).

### 2.5. Effects of the Composition and Culture Conditions of MRS Broth on Dominant Strain Growth

#### 2.5.1. The One Factor per Time (OFT) Approach

The one factor per time approach was used to optimize components of the original culture medium to achieve the highest growth of *P. pentosaceus* NCU006063. The effects of the carbon source, nitrogen source, trace elements, and growth factors and the best concentration from the carbon source, nitrogen source, trace elements, and growth factors on the growth of *P. pentosaceus* NCU006063 were first inspected by the OFT experimentation. One of the components of the original culture medium was removed in turn. All experiments were cultured in a culture medium at 37 °C for 24 h, and then the viable counts were determined. There were 3 parallel experiments in each group.

#### 2.5.2. Plackett–Burman Design (PBD)

Based on the results of the single-factor experiment, the Plackett–Burman experiment design method in Design-Expert 8.0 software was used to screen the above-influencing factors. PBD is an efficient linear model for screening the most important factors of fermentation parameters when many factors are available [24,25]. For screening carbon, nitrogen, trace elements, and growth factors, seven medium components were considered (maltose, soy peptone, Buffer salt, Tween 80, FeSO_4_, MnSO_4_·H_2_O, and VB7 for the design of PBD. Each factor was set at lower (−1) and higher levels (+1) and a total of 12 experimental groups were set (Table 1). Each experimental group was repeated three times, and the average viable counts were recorded. Finally, three factors (soy peptone, FeSO_4_, and VB_7_) contributing the most to the viable counts of the strain were selected for the steepest ascent experiment.

#### 2.5.3. Steepest Ascent Experiment

The steepest ascent experiment is relatively economical and can quickly determine the optimal area for each factor level. The effect value and change gradient of each factor were designed according to the results of the PB experiment [26]. According to the above results of the PB experiment, the steepest climbing experiment was carried out on the most significant factors for strain growth, and then the central region of the concentration value of the significant factors was determined.

#### 2.5.4. The Response Surface Analysis (RSM) Approach Utilizing the Central Composite Design (CCD) Technique

The center point and step size of the central composite design (CCD) experiment were determined based on the results of the steepest climb experiment [27,28], and the CCD test in the Design-Expert 8.0 software was used for RSM. The quadratic multiple regression fitting equations were established according to the model, and the results of the response surface diagram were generated. Then, the optimal ratio of significant factors in the medium components was obtained (Table 2).

#### 2.5.5. Effects of the MRS Broth Culture Conditions on Dominant Strain Growth

Based on the optimized MRS broth, the effects of three different incubation conditions, namely temperature (35 °C, 37 °C, 39 °C, 41 °C, 43 °C), initial pH (5, 5.5, 6, 6.5, 7, 7.5), and inoculum (1%, 2%, 3%, 4%, 5%) on the growth of *P. pentosaceus* NCU006063 were investigated separately using a single-factor experimental method. The viable counts were determined after incubation at 37 °C for 24 h. There were 3 parallel experiments in each group.

#### 2.5.6. Comparison of Growth Curves

*P. pentosaceus* NCU006063 was inoculated into MRS broth at 2% concentration and was cultured at 37 °C for 24 h. Meanwhile, *P. pentosaceus* NCU006063 was inoculated in the optimized medium according to the optimal inoculum volume and kept in the optimal culture conditions for 48h. The viable counts of the fermentation broth were measured at 0, 2, 4, 6, 8, 10, 12, 14, 16, 18, 20, 22, 24, 36, and 48 h. The growth curve was drawn to compare the two cultures so as to evaluate the influence of the optimized medium composition and culture conditions on the viable counts of *P. pentosaceus* NCU006063.

### 2.6. Optimization of Biomass Production Process Parameters

Based on determining the composition of the culture medium and culture conditions, the biomass production process parameters of *P. pentosaceus* NCU006063 were further optimized by using a 3.7L KLF fermenter produced by Biol Bioengineering Company in Wald, Switzerland. Constant pH (6.0, 6.5, 7.0), types of neutralizing agents (25% NH_3_·H_2_O, 25% NaOH, and 25% Na_2_CO_3_), and gas type (sterile air, nitrogen, and carbon dioxide) were selected for single factor experiments. After 24 h of culture, the viable number and OD_600_ of *P. pentosaceus* NCU006063 fermentation broth were determined, and the effects of different fermentation parameters were analyzed to determine the best fermentation parameters.

### 2.7. Statistical Analysis

All results were presented as the mean ± standard error of three independent experiments. The statistical analysis of the variance function of SPSS 27.0 for Windows software was used for statistical analysis. Statistical significance was established using Student’s *T*-test as *p* < 0.05.

## 3. Results

### 3.1. Strain Screening

#### 3.1.1. Determination of Acid Production Capacity

Twenty-four strains were selected from 12 factory-fermented cowpea samples to determine their acid production capacity (incubated at 37 °C for 48 h). Specifically, after 24 h incubation, the pH values of 10 isolates were <4 (Table S1 and Table 3), indicating high acid production capacity (12.97–18.78 g/kg) and meeting the requirements for factory cowpea fermentation. Overall, acidity increased with fermentation time, but the final acidity values of different strains were substantially different. Throughout the incubation period (48 h at 37 °C), the pH values of these 10 isolates continued to decrease. The pH values of 10 strains significantly reduced within the first 6 h after the peak. Remarkably, within the first 6 h, a severe decrease in pH value was observed in strain NCU006063, which is significantly different from other strains. This aligns with the TA values of these 10 isolates, which increased continuously during the whole incubation process (37 °C for 48 h). Interestingly, the TA content of NCU006063 was the highest, reaching 19.00 g/Kg. Hence, a total of 10 strains were selected based on their rapid acidification ability (pH < 4), and further analysis was conducted on their characteristics that may contribute to their use as probiotics in fermented cowpeas.

#### 3.1.2. Determination of Acid Tolerance Capacity

**Figure 1:**
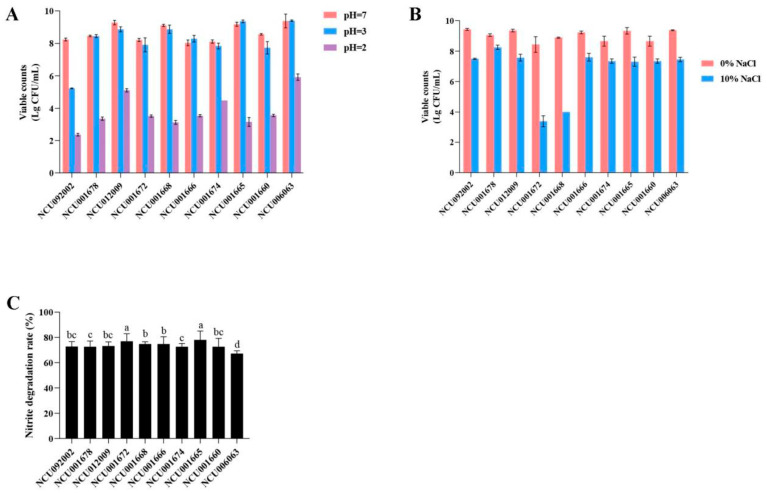
The acid tolerance, (**A**) salt tolerance, (**B**) degrade nitrite, and (**C**) capacity of lactic acid bacteria isolated from factory-fermentation samples was determined. Different lowercase letters (a, b, c, d) in the same graph indicate significant differences (*p* < 0.05).

As can be seen in Figure 1A, most of the tested strains were sensitive to acid. Except for *P. acidilactici* NCU092002, which had a low viable count (5.23 Log (CFU/mL)), all strains maintained high viability (7.84 Log (CFU/mL)) after 3 h at pH 3. However, after incubation at pH 2 for 3 h, only *P. pentosaceus* NCU006063 (5.92 Log CFU/mL) depicted the best viable count, while the other strains were lower than 5.11 Log (CFU/mL), indicating the sensitivity of these 9 remaining strains. In addition, *P. pentosaceus* NCU006063 showed significant survival rates, with the highest survival rate observed at pH 3 of 9.40 Log (CFU/mL).

#### 3.1.3. Determination of Salt Tolerance Capacity

Figure 1B shows that after 24 h (37 °C) of cultivation in MRS broth containing 10% NaCl, the growth of 10 strains was inhibited to varying degrees. Except for strains NCU001672 and NCU001668, with viable bacterial counts below 4 Log (CFU/mL), the viable bacterial counts of the other 8 strains were increased, reaching 7 Log (CFU/mL). These 8 strains may have high osmotic pressure resistance and high tolerance to acid.

#### 3.1.4. Determination of Degrade Nitrite Capacity

The results of the influence of lactic acid bacteria on nitrite degradation are shown in Figure 1C. The results showed that after incubation at 37 °C for 24 h, the content of sodium nitrite decreased, which indicates that each strain has a specific ability to degrade nitrite, with a degradation rate of 67.17–78.01%. In fact, *L. plantarum* NCU001674 displayed the highest nitrate degradation rate (78.01%). This may be due to the varying activity of nitrite reductase in different strains [29]. Consistently, some lactic acid bacteria have nitrite reductase systems capable of reducing nitrite to nitrogen dioxide, nitrous oxide, or nitrogen under anaerobic conditions [30]. Lactic acid bacteria can degrade 61.4–92.7% of nitrite after 24 h at 30 °C under anaerobic conditions. These results clearly showed that the strain isolated from cowpea was highly influential in consuming sodium nitrite when incubated at 37 °C for 24 h.

#### 3.1.5. Principal Component Analysis of Bacterial Strain Properties

**Figure 2:**
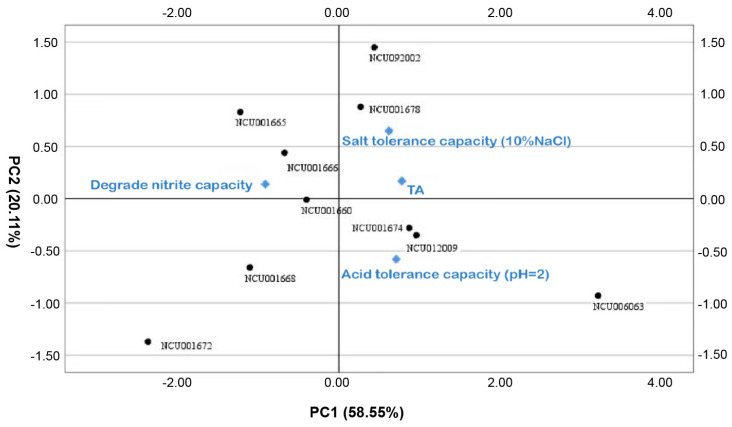
The biplot of principal component analysis based on LAB capacities.

Principal component analysis was performed to explore the similarities and differences in LAB from various characteristics. Mainly three principal components (PC) were involved, accounting for 94.61% of the total variation (Table S2). PC1 explained 58.55% of the variation, characterized by its ability to degrade nitrite. PC2 explained 20.11% of the variation achieved by salt tolerance. PC3 explained 15.95% of the variation, characterized by acid production. The correlation and contribution of LAB probiotic characteristics could be obtained from the biplot in Figure 2. In order to further comprehensively and intuitively evaluate the probiotic performance of each LAB, four variables were normalized and multiplied with the component matrix to obtain a comprehensive score. As shown in Table 4, the probiotic with the highest score was *P. pentosaceus* NCU006063, which can be used as the target LAB.

### 3.2. Effects of the Compositions and Culture Conditions of MRS Broth on P. pentosaceus NCU006063 Growth

#### 3.2.1. Effects of the Compositions of MRS Broth on *P. pentosaceus* NCU006063 Growth

**Figure 3:**
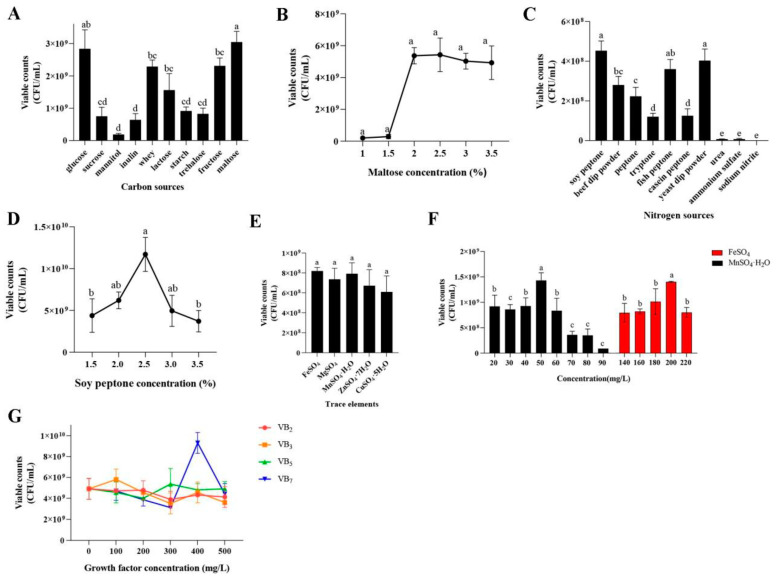
Effects of the carbon sources (**A**) maltose concentration, (**B**) nitrogen sources, (**C**) soy peptone concentration, (**D**) trace elements, (**E**) trace elements concentration, and (**F**) growth factor types and concentration on (**G**) *P. pentosaceus* NCU006063 growth. Different lowercase letters (a, b, c, d, e) in the same graph indicate significant differences (*p* < 0.05).

As shown in Figure 3A, the optimal carbon source for the growth of *P. pentosaceus* NCU006063 was maltose with a viable bacterial count of (3.05 × 10^9^ CFU/mL) (Figure 3A). Therefore, maltose was chosen as the sole carbon source for growth. In addition, the number of *P. pentosaceus* NCU006063 first increased with the increase in maltose concentration and then remained unchanged. Therefore, the contents of maltose 2.0% and 2.5% were the most suitable carbon source for the growth of *P. pentosaceus* NCU006063 with a viable bacterial count of 5.37 × 10^9^ CFU/mL (Figure 3B). To save costs, we selected 2.0% maltose with a viable bacterial count (5.37 × 10^9^ CFU/mL) (Figure 3B). The effect of the nitrogen source on the growth of *P. pentosaceus* NCU006063 was investigated at the optimum medium carbon source. As shown in Figure 3C, different nitrogen sources significantly impacted bacterial growth (*p* < 0.05). Compared with other nitrogen sources, soybean peptone has higher bacterial growth, so further optimization is needed. In addition, when the soy peptone content was 2.5%, the number of bacteria first increased and then decreased with the increase in lactose concentration, and the bacterial growth was the highest (1.17 × 10^10^ CFU/mL).

Further, the effect of salt ions on the growth of *P. pentosaceus* NCU006063 was investigated at the medium’s optimum carbon sources and nitrogen sources. Although there were no significant differences in the effect of FeSO_4_, MgSO_4_, MnSO_4_·H_2_O, ZnSO_4_·7H_2_O, and CuSO_4_·5H_2_O on the growth of *P. pentosaceus* NCU006063 (Figure 3E), FeSO_4_ and MnSO_4_·H_2_O were slightly higher than other salt ions. Therefore, FeSO_4_ and MnSO_4_·H_2_O were selected for salt ions, and the *P. pentosaceus* NCU006063 showed the highest growth when the concentrations of FeSO_4_ and MnSO_4_·H_2_O were 200 mg/L (1.44 × 10^9^ CFU/mL) and 50 mg/L (1.43 × 10^9^ CFU/mL) (Figure 3F). Based on the optimum salt ions, carbon sources, and nitrogen sources of the medium, VB_7_ is the optimal bacterial growth and the optimal growth concentration is 400 mg/L (9.30 ± 0.90) ×10^9^) (Figure 3G).

#### 3.2.2. Screening of Significant Variables Using Plackett–Burman Design

To improve the vital viability of *P. pentosaceus* NCU006063, the PBD was used to select the most significant factors. The viability of *P. pentosaceus* NCU006063 varied widely among the 12 experiments, ranging from 8.15 to 9.98 Lg (CFU/mL) (Table S3).

Table 5 presents the statistical analysis of PBD experimental data. It was found that soy peptone (*p*-values = 0.0260), FeSO_4_ (*p*-values = 0.0028), and VB_7_ (*p*-values = 0.0191) were the most effective in the growth of the *P. pentosaceus* NCU006063. Subsequently, the substantial steepest ascent experiment determined the optimal concentration of each specific factor.

#### 3.2.3. Steepest Ascent Experiment

The steepest ascent experiment was performed to determine the maximum viable counts of *P. pentosaceus* NCU006063. As shown in Table 6, the viable counts of *P. pentosaceus* NCU006063 gradually increased (6.15 × 10^9^ CFU/mL) before experiment 4. They then gradually decreased, indicating that the optimal culture conditions for *P. pentosaceus* NCU006063 were close to the conditions of experiment 4. Therefore, the conditions of experiment 4 were selected for further optimization, and their interactions were further analyzed.

#### 3.2.4. Center Combination Design (CCD)

The results of CCD experiments to study the effect of soy peptone, FeSO_4,_ and VB_7_ on the viable counts of *P. pentosaceus* NCU006063 are shown in Table S4, while Table S5 depicts the statistical analysis of results. According to the current model, the interaction between soy peptone and FeSO_4_, as well as the two squared models (soy peptone, A^2^; FeSO_4_, B^2^; VB_7_, C^2^), have a significant effect on the number of *P. pentosaceus* NCU006063 (*p* < 0.05). Analysis of variance (ANOVA) showed that *p*-value = 0.0005, indicating the significance of the regression model. The R^2^ of the regression equation was 0.9067, indicating that the model could explain 90.67% of the response variability. A second-order polynomial mathematical model for the viable counts of *P. pentosaceus* NCU006063 was proposed, including different interactions of high and low levels of various factors.

**Figure 4:**
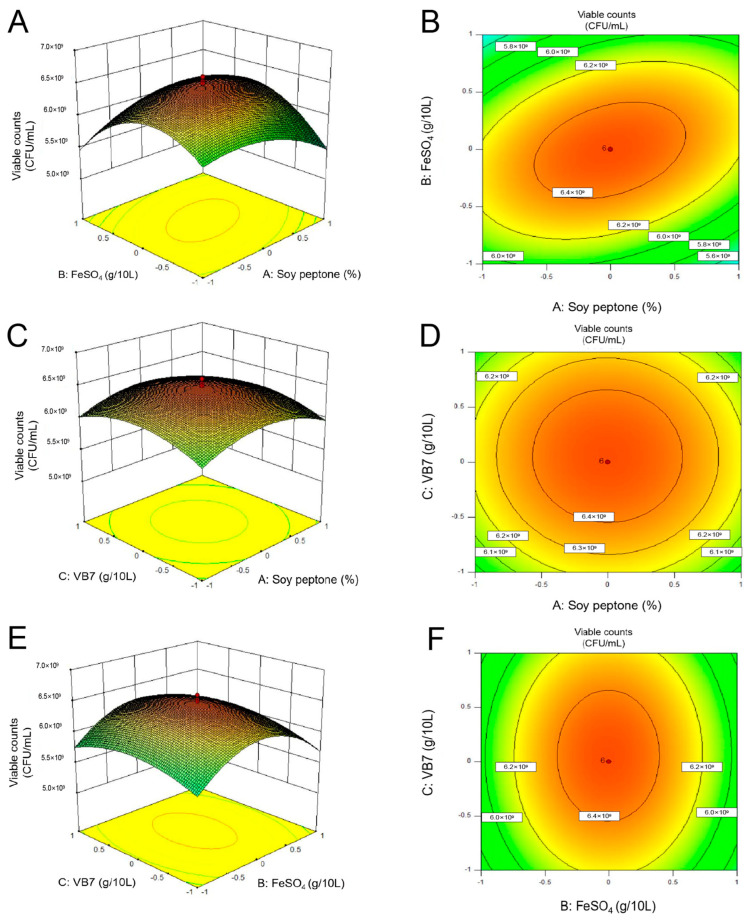
Response surface plots and corresponding contour plots of the interaction of the three factors. ((**A**): Response surface plot of FeSO_4_ and soy peptone interaction, (**B**): Contour plot of FeSO_4_ and soy peptone interaction; (**C**): Response surface plot of VB_7_ and soy peptone interaction, (**D**): Contour plot of VB_7_ and soy peptone interaction; (**E**): Response surface plot of VB_7_ and FeSO_4_ interaction, (**F**): Contour plot of VB_7_ and FeSO_4_ interaction).

Response surface plot and contour plot of the pairwise interaction of three factors affecting the viable counts of *P. pentosaceus* NCU006063 were shown in Figure 4. The data obtained indicated that the use of soy peptone, FeSO_4_, and VB_7_ resulted in an increase in viable counts of *P. pentosaceus* NCU006063 at optimal levels. The maximum points of the model were as follows: soy peptone (A) 38.75 g/L, FeSO_4_ (B) 0.10 g/L, and VB_7_ (C) 20 g/L, respectively. At this point, the maximum viable counts of *P. pentosaceus* NCU006063 were 7.92 × 10^9^ CFU/mL. In order to verify the prediction results of the model, a validation experiment is carried out under the predicted conditions. The observed value (7.90 × 10^9^ CFU/mL) was quite close to the expected value, which confirmed the validity of the response model and the existence of the optimal point.

#### 3.2.5. Effects of the MRS Broth Culture Conditions on *P. pentosaceus* NCU006063 Growth

**Figure 5:**
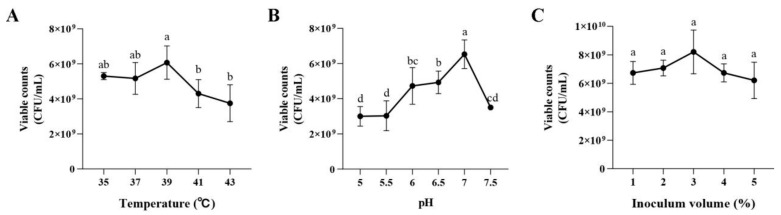
Effects of temperature (**A**) pH (**B**) and inoculum volume (**C**) on the growth of *P. pentosaceus* NCU006063. Different lowercase letters (a, b, c, d) in the same graph indicate significant differences (*p* < 0.05).

Figure 5A illustrates the number of live bacteria in the LAB after incubation at different temperatures for 24 h. An inflection point was found at 39 °C, indicating that this temperature (6.07 × 10^9^ CFU/mL) was the optimal culture for *P. pentosaceus* NCU006063. Figure 5B showed that excessively high or low pH inhibited the activity of *P. pentosaceus*, with pH 7 (6.53 × 10^9^ CFU/mL) being the best initial pH of *P. pentosaceus* NCU006063. Then, the effect of inoculum volume on the activity of *P. pentosaceus* was within 1–3%, and the viable counts of LAB increased with the increase in the inoculum volume. When the inoculum volume exceeded 3%, the viable counts of LAB began to decline (Figure 5C). Therefore, the optimal inoculum volume of *P. pentosaceus* NCU006063 was 3%, with a viable count of 8.20 × 10^9^ CFU/mL. After all, the optimal fermentation temperature, initial pH value, and inoculum volume were 39 °C, 7 and 3%, respectively.

#### 3.2.6. Comparison of the Growth Curves of *P. pentosaceus* NCU006063

**Figure 6:**
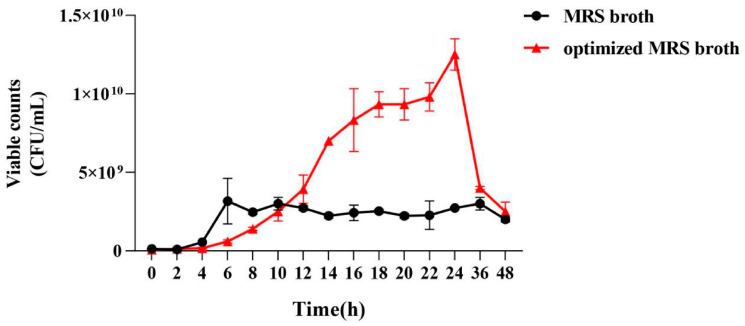
Effect of optimized medium compositions and culture conditions on the viable counts of *P. pentosaceus* NCU006063.

From Figure 6, *P. pentosaceus* NCU006063 entered the logarithmic growth period after 4 h of incubation in the MRS broth, and the viable counts increased rapidly. After incubation for 6 h, it reached a stable stage, and the number of viable bacteria did not show significant changes. In contrast, the logarithmic growth period of *P. pentosaceus* NCU006063 was delayed to the 6th h, and the stabilization phase was postponed to the 18th h. Compared with MRS broth (2.73 × 10^9^ CFU/mL), the growth rate of optimized *P. pentosaceus* NCU006063 was significantly increased. At 24 h, the number of viable counts of optimized *P. pentosaceus* NCU006063 was 1.25 × 10^10^ CFU/mL, which was 4.58 times higher than that in MRS broth (2.73 × 10^9^ CFU/mL).

### 3.3. Optimization of Biomass Production Process Parameters

**Figure 7:**
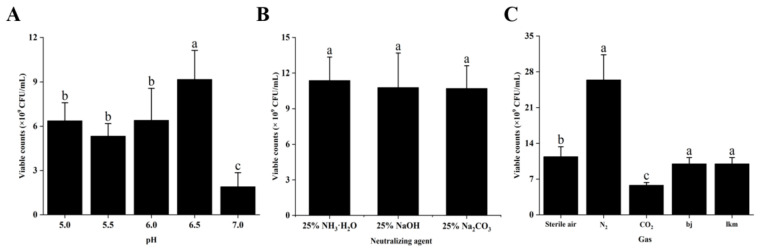
Constant pH (**A**) types of neutralizing agents (**B**) and gas type (**C**) on the growth of *P. pentosaceus* NCU006063. Different lowercase letters (a, b, c) in the same graph indicate significant differences (*p* < 0.05).

Figure 7A shows that the total number of viable bacteria of *P. pentosaceus* NCU006063 tends to increase first and then decrease with increasing pH after 24 h of cultivation. The maximum number of viable bacteria was observed at pH 6.5 (9.17 × 10^9^ CFU/mL), indicating that this is the optimal constant pH value for culturing *P. pentosaceus* NCU006063. In addition, Figure 7B summarizes the number of viable bacteria under different neutralizing agents (25% NH_3_·H_2_O, 25% NaOH, 25% Na_2_CO_3_), with 25% NH_3_·H_2_O (1.14 × 10^10^ CFU/mL) being the optimal protective agent for pentose NCU006063. Likewise, Figure 7C displayed the number of viable bacteria cultured in sterile air, nitrogen (N_2_), and carbon dioxide (CO_2_) after 24 h. The number of viable bacteria under N_2_ (2.65 × 10^10^ CFU/mL) is much higher than the two other LAB strains. After all, the constant pH, neutralizing agent types, and gas types were 6.5, 25%NH_3_·H_2_O and N_2_, respectively. At this point, the number of viable bacteria reached 2.65 × 10^10^ CFU/mL, which is 9.71 times the number of viable bacteria in the MRS medium.

## 4. Discussion

Spontaneous fermentation mainly utilizes the high permeability of salt and microorganisms attached to the surface of vegetables during the fermentation process. It has a long fermentation cycle, unstable safety, and was prone to harmful microbial contamination [31]. Direct fermentation of vegetables with lactic acid bacteria could increase the total acid quickly, accelerate the fermentation speed, shorten the fermentation time, and result in low nitrite content in the finished product. Meanwhile, the growth of beneficial microorganisms could inhibit the abnormal fermentation caused by bacterial infection and growth, thereby improving the safety of fermented products to a certain extent [32]. Therefore, to promote the broader application of lactic acid bacteria fermentation in cowpeas, this study conducted screening of good fermentation agents and biomass production of cowpea lactic acid bacteria fermentation agents.

Lactic acid produced by lactic acid bacteria (LAB), such as *Lactobacillus pentosus* [33] and *Lactobacillus acidophilus* [34], inhibited the growth of miscellaneous bacteria while screening strains under more acidic growth conditions. The screening process was primarily directed at the ability of the strains to produce lactic acid [35,36]. In this study, 10 of the 24 strains tested achieved pH < 4 after 24 h of culture, and their corresponding TA ranged from 12.97 g/kg to 17.17g/kg. It found that when *Lactobacillus paracasei* was used as the starter to ferment Chinese Cabbage (Brassica rapa var. pekinensis), the pH was lower than 4, and the lactic acid content was 7.13 g/L [37]. Consistently, these 10 isolated strains may have potential applications in fermented cowpeas.

Furthermore, as is known to all, LAB strains survived at pH 4 [38], which was also a typical final ripening acidity in many fermented vegetables [39,40]. Although the survival rate of most strains significantly decreased after exposure to pH 2 for 3 h, all strains tested were tolerant after 3 h of incubation at pH 3. This is consistent with previous findings that LAB strain maintained viability at pH 2.5 to 4.0, but lost viability at lower pH values [41,42]. In particular, the *P. pentosaceus* NCU006063 was the most potent acidifier, with the fastest pH decline and the highest TA production. Except for strains NCU001672 and NCU001668, the other 8 strains showed good salt tolerance, indicating that they are highly resistant to osmotic pressure.

Meanwhile, nitrate accumulation was a common problem affecting fermented vegetable production and consumer health, including cowpea [43], black olives [44], and paocai [45]. Previous research reports have suggested that nitrite reduction in fermented vegetables during fermentation is due to the degradation of lactic acid bacteria [46]. Excessive intake of nitrite can lead to fatal diseases, such as methemoglobinopathy and stomach cancer [47,48]. Therefore, the ability of lactic acid bacteria to degrade nitrite is an important indicator for screening cowpea fermentation. In terms of the ability of the strains to degrade nitrite, each strain had some ability, and the difference in degradation rate was minimal (65.17–78.01%) in this study. The difference in nitrite degradation rates may be due to the varying activity of nitrite reductase in different strains. The nitrite degradation rates of 275 lactic acid bacteria isolates were between 4.8% and 99.9%, with 50% showing good nitrite degradation capability, averaging over 91.3% [49]. It has also been demonstrated that some lactic acid bacteria possess nitrite reductase systems, which can reduce nitrite to nitrogen dioxide, nitrous oxide, or nitrogen under anaerobic conditions. This indicated that the strain isolated from the cowpea was very efficient in consuming nitrite when incubated at 37 °C for 24 h.

Since principal component analysis (PCA) can be used to select the optimal choice from multiple indicators in objective analysis, it was widely performed to select more promising probiotic candidates from numerous indicators [50,51,52]. The final score, taking the four characteristics, including acid production, acid tolerance, salt tolerance, and nitrite denaturation rate of the strains, was calculated through PCA. Finally, *P. pentosaceus* NCU006063 was ultimately selected as the inoculant to produce starting cultures with ideal characteristics.

In addition, maltose is a carbon source and an inducer. As a carbon source, it accelerates the growth of bacteria and significantly shortens the time from thallus growth to the logarithmic phase [53]. The results showed that the growth of *P. pentosaceus* NCU006063 was closely related to the type of carbon source. Here, maltose (2%) was the best carbon source for the growth of *P. pentosaceus* NCU006063. As reported previously by Liu et. (2018), the use of maltose as a carbon source promotes the growth of LABs, such as *Bifidobacterium* and *Lactobacillus acidophilus* [54]. As an inducer, it provoked the secretion and expression of related enzymes during the growth of bacteria [55]. Accordingly, soy protein was considered as an ideal nitrogen source for certain LAB [56]. With respect to these previous findings, our results presented the highest viable bacterial count, which could be achieved at a nitrogen source of 2.5% (1.17 × 10^10^ CFU/mL), indicating soy peptone could serve as the sole nitrogen source for certain probiotics.

In addition, FeSO_4_ and MnSO_4_·H_2_O have positively affected the growth of *P. pentosaceus* NCU006063. The reason might be that iron and manganese ions were beneficial to developing *P. pentosaceus* NCU006063. Often, manganese ions was a component of essential enzymes involved in glucose metabolism [57]. Therefore, appropriate amounts could provide more ATP for lactate synthesis in glucose metabolism.

VB_7_ is essential for the normal metabolism of fats, proteins, and carbohydrates and can promote cell growth and metabolism. Previous studies have found that VB_7_ could increase the thickness of bacterial biofilms, as well as the content of extracellular polysaccharides, proteins, and lipids [58]. Therefore, when VB7 is used as a growth factor, the activity of *P. pentosaceus* NCU006063 was seriously increased.

Plackett–Burman design (PBD) was particularly suitable when there are many factors present, and the significance of each factor relative to the response variable has not yet been determined. Then, the steepest ascent experiment could be used to determine the central point of the response surface analysis to optimize the experimental data. Finally, response surface experiments could be conducted while considering multiple factors and their interactions to better understand the relationship between response variables and factors. These design methods could better control experiment variables and evaluation factors, thereby more accurately evaluating experimental results [59,60,61]. PBD, obstacle ascent experiment, and CCD were used to obtain a modified optimized MRS broth. By optimizing the MRS broth culture conditions and biomass production process parameters, the viable counts of *P. pentosaceus* NCU006063 could be received in the optimized MRS broth, with viable counts of 2.65 × 10^10^ CFU/mL during 24 h of incubation.

Many microorganisms in the fermentation process are rich in enzymes that were essential for flavor formation, such as *Acinetobacter*, *Enterobacter*, *Raoultella*, *Enterococcus*, *Klebsiella*, *Lactococcus*, *Leuconostoc*, *Weissella*, *Lactiplantibacillus*, and *Limosilactobacillus*, all of which function to varying degrees in the flavor of fermented bamboo shoots [11]. The fermentation process also produced many toxic substances, such as biogenic amines, which were mainly produced by decarboxylation of free amino acids by specific microorganisms using decarboxylase enzymes [62], such as *Enterobacter, Vibrio, and Staphylococcus, Salmonella, Lactobacillus* [63]. Lactobacillus was a common decarboxylase-producing bacterium [64,65]. Further assessment of the benefits and disadvantages of microorganisms in fermented cowpea can be subsequently built on this basis.

In a nutshell, this study identified promising strains of lactic acid bacteria as a fermentation agent for cowpeas fermentation. The optimized composition of nitrogen sources in MRS broth has been dramatically simplified, reducing costs, optimizing growth conditions, and providing a safer, cheaper, and simpler future factory production method.

## 5. Conclusions

In short, four characteristics of strains were comprehensively considered, including acid production, acid resistance, salt tolerance, and nitrite degradation rate. Finally, *P. pentosaceus* NCU006063 was selected as the inoculum strain to produce a starting culture with ideal characteristics. Soybean peptone, FeSO_4,_, and VB_7_ were important factors affecting the number of viable bacteria in *P. pentosaceus* NCU006063. Herein, we developed an optimized MRS broth with a single nitrogen source and high viable counts. Over time, *P. pentosaceus* NCU006063 depicted a high number of viable bacteria in the modified MRS broth containing maltose, soybean peptone, triamine citrate, anhydrous dipotassium phosphate, anhydrous sodium acetate, Tween 80, ferric sulfate, manganese sulfate monohydrate, and VB_7_. The conditions of cultures, such as temperature (39 °C), initial pH (7.0), and inoculation amount (3%), as well as optimized biomass production parameters with constant pH (6.5), neutralizing agent types (25% NH_3_·H_2_O), and gas types (N_2_) were most suitable as fermentation agents for *P. pentosaceus* NCU006063. Overall, the present study was beneficial in improving the quality and viable bacterial counts of fermented cowpeas and contributing to the production of high-quality fermented cowpeas. Subsequently, specific analyses of microorganisms in fermented cowpeas can be carried out to explore in depth the influence of microbial communities on the flavor and safety of fermented cowpeas during fermentation and to provide more theoretical basis for fermenting cowpeas in factories.

## Figures and Tables

**Table 1:** Range of different factors investigated with Plackett–Burman.

Variable Code	Variable	Low Level (−1)	High Level (+1)
A	Maltose (g/L)	10	30
B	Soy peptone (g/L)	15	35
C	Buffer salt (g/L)	5.16	7.16
D	Tween 80 (g/L)	1	2
E	FeSO_4_ (mg/L)	100	300
F	MnSO_4_·H_2_O (mg/L)	40	60
G	VB_7_ (mg/L)	300	500

**Table 2:** Range of different factors investigated with the central composite design (CCD).

Variable Code	Variable	Level				
		−1.682	−1	0	1	1.682
A	Soy peptone (%)	3.34	3.5	4.0	4.5	4.66
B	FeSO_4_ (g/10 L)	0.71	0.8	1.1	1.4	1.49
C	VB_7_ (g/10 L)	1.84	2.0	2.5	3.0	3.16

**Table 3:** The acid production capacity of lactic acid bacteria isolated from factory-fermentation samples was determined.

	NCU092002	NCU001678	NCU012009	NCU001672	NCU001668	NCU001666	NCU001674	NCU001665	NCU001660	NCU006063
	*P. acidilactici*	*L. plantarum*	*L. paracasei*	*L. plantarum*	*L. plantarum*	*L. plantarum*	*L. plantarum*	*L. plantarum*	*L. plantarum*	*P. pentosaceus*
pH										
0 h	6.23	6.23	6.23	6.23	6.23	6.23	6.23	6.23	6.23	6.23
2 h	5.93	5.82	5.72	5.88	5.69	5.48	5.81	5.42	5.56	4.62
4 h	5.49	5.47	5	5.57	5.15	5.53	5.66	4.89	5.2	4.39
6 h	5.02	4.58	4.39	4.67	4.36	4.62	4.77	4.1	4.43	4.24
8 h	4.7	4.1	4.16	4.33	4.02	4.21	4.39	3.98	4.13	4.05
10 h	4.49	3.99	3.96	4.12	3.94	4.04	4.19	3.79	4.03	4.03
12 h	3.84	3.84	3.84	3.99	3.84	3.95	3.93	3.79	3.9	3.99
24 h	3.67	3.71	3.71	3.83	3.67	3.76	3.69	3.7	3.73	3.84
36 h	3.92	3.73	3.78	3.83	3.72	3.74	3.67	3.74	3.68	3.8
48 h	3.85	3.66	3.8	3.72	3.6	3.64	3.62	3.65	3.63	3.87
TA (g/kg)										
0 h	0.17 ± 0.82	0.17 ± 0.00	0.17 ± 0.82	0.17 ± 0.00	0.17 ± 0.00	0.17 ± 0.00	0.17 ± 0.00	0.17 ± 0.00	0.17 ± 0.00	0.17 ± 0.00
2 h	2.31 ± 0.66	2.31 ± 0.66	3.20 ± 0.76	2.49 ± 0.48	2.49 ± 0.48	2.31 ± 0.66	2.13 ± 0.84	3.02 ± 0.94	2.67 ± 0.03	2.31 ± 0.66
4 h	3.56 ± 0.04	3.02 ± 0.94	4.98 ± 0.96	2.67 ± 0.03	3.38 ± 0.58	2.67 ± 0.03	2.49 ± 0.48	4.09 ± 0.86	3.20 ± 0.76	5.88 ± 0.06
6 h	4.81 ± 0.14	4.81 ± 0.14	9.08 ± 0.82	6.05 ± 0.88	6.59 ± 0.34	6.23 ± 0.07	4.81 ± 0.14	8.19 ± 0.72	6.23 ± 0.07	7.48 ± 0.44
8 h	6.59 ± 0.34	8.37 ± 0.54	11.04 ± 0.84	6.94 ± 0.98	9.80 ± 0.01	7.84 ± 0.08	6.59 ± 0.34	9.97 ± 0.92	8.37 ± 0.54	8.91 ± 0.00
10 h	7.84 ± 0.08	9.62 ± 0.28	12.65 ± 0.22	8.19 ± 0.72	10.33 ± 1.35	9.08 ± 0.82	8.01 ± 0.09	11.04 ± 0.84	9.08 ± 0.82	10.3 ± 0.35
12 h	8.350 ± 0.56	12.65 ± 0.22	13.22 ± 0.17	10.87 ± 0.02	13.54 ± 0.32	11.58 ± 0.03	11.40 ± 0.48	14.07 ± 0.78	12.65 ± 0.22	11.76 ± 0.12
24 h	13.04 ± 0.77	15.89 ± 0.49	18.78 ± 0.87	12.97 ± 0.17	16.62 ± 0.57	14.06 ± 0.79	16.26 ± 0.03	15.89 ± 0.49	16.26 ± 0.03	15.71 ± 0.22
36 h	16.17 ± 0.92	15.71 ± 0.22	21.39 ± 0.83	14.25 ± 0.06	17.17 ± 0.38	15.34 ± 0.68	17.53 ± 0.92	16.07 ± 0.76	17.35 ± 0.65	17.90 ± 0.46
48 h	18.78 ± 0.87	16.62 ± 0.57	17.20 ± 0.37	14.98 ± 0.14	17.17 ± 0.38	15.52 ± 0.95	17.17 ± 0.38	16.44 ± 0.03	15.52 ± 0.95	19.00 ± 0.08

**Table 4:** Comprehensive scores obtained from principal component analysis of LAB strains.

Strains	Y1	Y2	Y3	Y
NCU006063	3.21	−0.93	−0.29	1.64
NCU012009	0.96	−0.35	0.58	0.59
NCU001674	0.87	−0.28	0.37	0.51
NCU001678	0.27	0.88	0.4	0.4
NCU092002	0.44	1.45	−1.39	0.33
NCU001660	−0.4	−0.01	0.6	−0.14
NCU001666	−0.67	0.44	0.91	−0.16
NCU001665	−1.22	0.83	0.29	−0.5
NCU001668	−1.1	−0.66	−1.34	−0.99
NCU001672	−2.36	−1.37	−0.14	−1.68

**Table 5:** Statistical analyses of Plackett–Burman design showing the calculated regression coefficient, standard error t and *p*-values for each variable for viable counts of *P. pentosaceus* NCU006063.

Factors	Coefficient Estimates	Standard Error	SS	MS	t-Values	*p*-Values
Intercept	9.1308	0.0521	2.69	0.38	11.79	0.0156 *
Maltose	0.0066	0.0521	0.0005	0.0005	0.016	0.9047
Soy peptone	0.1798	0.0521	0.39	0.39	11.92	0.0260 *
Buffer salt	0.1108	0.0521	0.15	0.15	4.53	0.1004
Tween 80	−0.0642	0.0521	0.05	0.05	1.52	0.285
FeSO_4_	−0.3403	0.0521	1.39	1.39	42.71	0.0028 **
MnSO_4_·H_2_O	0.1415	0.0521	0.24	0.24	7.38	0.0532
VB_7_	−0.1979	0.0521	0.47	0.47	14.45	0.019 *

* *p*-values less than 0.05 indicated that the model terms are significant, ** *p*-values less than 0.01 indicated that the model terms are significant. R^2^ = 0.9538, R^2^Adj = 0.8729, Adeq Precision = 11.672, C.V. % = 1.98. SS: sum of squares, MS: mean square.

**Table 6:** The steepest rise experiment was used to determine the optimal concentration of a specific factor.

Experiment	Soy Peptone (%)	FeSO_4_ (g/10 L)	VB_7_ (g/10 L)	Viable Counts (×10^9^ CFU/mL) ^a^
1	2.5	2.0	4.0	3.50
2	3.0	1.7	3.5	3.33
3	3.5	1.4	3.0	4.60
4	4.0	1.1	2.5	6.15
5	4.5	0.8	2.0	5.73
6	5.0	0.5	1.5	3.30

^a^ Values listed are the averages of three experiments.

## Data Availability

The original contributions presented in the study are included in the article/Supplementary Material, further inquiries can be directed to the corresponding author.

## Supplementary Materials

The following supporting information can be downloaded at: https://www.mdpi.com/article/10.3390/foods14020150/s1, Table S1: The closest related type strains of sequences of isolates from fermented cowpea in NCBI Genbank; Table S2: The component matrix and eigenvectors of LAB properties obtained by principal component analysis; Table S3: Plackett-Burman design matrix for assessing variables affecting the viability of *P. pentosaceus* NCU006063; Table S4: The results of CCD experiments to study the effect of soy peptone, FeSO4 and VB7 on the viable counts of *P. pentosaceus* NCU006063; Table S5: The statistical analysis of CCD experiments results.
