# Supplementary material for: Screening of Lactic Acid Bacteria Isolated from Fermented Cowpea and Optimization of Biomass Production Conditions
Source: Foods. 2025 Jan 7;14(2):150. doi: 10.3390/foods14020150 (PMC11765374; doi:10.3390/foods14020150)
Supplement: Supplementary file 1 [file foods-14-00150-s001.zip › foods-3365612-supplementary.pdf]

Table S1 The closest related type strains of sequences of isolates from fermented cowpea in NCBI Genbank

isolates	closest relative sequence	Accession number[a]	Length（bp）	Similarity (%)
NCU092002	*Pediococcus acidilactici strain* MBO002	MT007289.1	1196	98.39
NCU001678	*Lactobacillus plantarum strain* 2180	MT597730.1	1205	97.57
NCU012009	*Lactobacillus paracasei strain* 4613	MT545108.1	1197	97.82
NCU001672	*Lactobacillus plantarum strain* 2180	MT604680.1	1178	99.06
NCU001668	*Lactobacillus plantarum strain* 1944	MT597760.1	1187	98.31
NCU001666	*Lactobacillus plantarum strain* 5645	MT510468.1	1142	98.51
NCU001674	*Lactobacillus plantarum strain* 5585	MT510328.1	1179	98.05
NCU001665	*Lactobacillus plantarum strain* 1911	MT597730.1	1198	97.91
NCU001660	*Lactobacillus plantarum strain* 6415	MT515856.1	1211	97.91
NCU006063	*Pediococcus pentosaceus strain* 6340	MT463749.1	1175	97.7

[a] Accession number of the sequence of the closest relative found by BLAST search.

Table S2 The component matrix and eigenvectors of LAB properties obtained by principal component analysis.

Varibles	Component matrix			Eigenvectors		
	PC1 (58.27%)	PC2 (20.29%)	PC3 (16.02%)	PC1	PC2	PC3
TA (48h)	0.779	0.173	-0.566	0.509	0.192	-0.708
acid tolerance capacity （PH=3）	0.714	-0.576	0.345	0.467	-0.640	0.431
salt tolerance capacity （10% NaCl）	0.618	0.651	0.438	0.404	0.723	0.548
degrade nitrite capacity	-0.919	0.137	0.083	-0.601	0.152	0.104

Table S3 Plackett-Burman design matrix for assessing variables affecting the viability of *P. pentosaceus* NCU006063.

Run	A	B	C	D	E	F	G	Viable counts（Lg CFU/mL）[a]
1	-1	1	1	-1	1	1	1	8.93
2	1	-1	1	1	-1	1	1	9.40
3	-1	-1	-1	-1	-1	-1	-1	9.30
4	-1	-1	1	-1	1	1	-1	9.27
5	1	1	1	-1	-1	-1	1	9.54
6	-1	1	-1	1	1	-1	1	8.64
7	1	-1	1	1	1	-1	-1	8.64
8	-1	1	1	1	-1	-1	-1	9.67
9	-1	-1	-1	1	-1	1	1	8.94
10	1	-1	-1	-1	1	-1	1	8.15
11	1	1	-1	-1	-1	1	-1	9.98
12	1	1	-1	1	1	1	-1	9.11

[a] Values listed are the averages of three experiments. A: maltose, B: Soy peptone, C: buffer salt, D: Tween 80, E: $FeSO_4$, F: $MnSO_4 \cdot H_2O$ and G: VB_7.

Table S4 The results of CCD experiments to study the effect of soy peptone, $FeSO_4$ and VB_7 on the viable counts of *P. pentosaceus* NCU006063.

Run	Variable code			Viable counts ($x10^9$ CFU/mL) [a]
	A	B	C	
1	0	0	-1.68	6.05
2	1	-1	-1	5.20
3	0	0	0	6.50
4	-1	-1	-1	5.47
5	0	0	0	6.60
6	-1.68	0	0	5.70
7	-1	1	-1	5.25
8	-1	1	1.	5.15
9	0	0	0	6.43
10	0	-1.68	0	5.07
11	1	-1	1	5.07
12	0	0	0	6.53
13	0	0	1.68	5.80
14	0	0	0	6.60
15	1.68	0	0	5.96
16	0	1.68	0	5.13
17	1	1	1	5.80
18	0	0	0	6.20
19	1	1	-1	5.30
20	-1	-1	1	5.97

[a] Values listed are the averages of three experiments. A: Soy peptone, B: $FeSO_4$, C: VB_7.

Table S5 The statistical analysis of CCD experiments results.

Source	SS	DF	MS	t-values	p-values
Model	$5.40x10^{18}$	9	$6.00x10^{17}$	10.7978	0.0005 **
A	$4.15x10^{13}$	1	$4.15x10^{13}$	0.0007	0.9787
B	$5.66x10^{14}$	1	$5.66x10^{14}$	0.0102	0.9216
C	$8.78x10^{15}$	1	$8.78x10^{15}$	0.1579	0.6995
AB	$4.36x10^{17}$	1	$4.36x10^{17}$	7.8334	0.0188 *
AC	$1.39x10^{14}$	1	$1.39x10^{14}$	0.0025	0.9611
BC	$1.39x10^{14}$	1	$1.39x10^{14}$	0.0025	0.9611
A^2	$1.00x10^{18}$	1	$1.00x10^{18}$	18.0136	0.0017 **
B^2	$3.93x10^{18}$	1	$3.93x10^{18}$	70.7081	$<$0.0001 **
C^2	$7.67x10^{17}$	1	$7.67x10^{17}$	13.7862	0.0040 **
Residual	$5.56x10^{17}$	10	$5.56x10^{16}$		
Lack of Fit	$4.43x10^{17}$	5	$8.87x10^{16}$	3.9384	0.0794
Pure Error	$1.13x10^{17}$	5	$2.25x10^{16}$		
Cor Total	$5.96x10^{18}$	19			

* p-values less than 0.05 indicated that the model terms are significant, ** p-values less than 0.01 indicated that the model terms are significant. A: Soy peptone, B: $FeSO_4$, C: VB_7. R^2 = 0.9067, R^2Adj=0.8227, Adeq Precision=8.925, C.V. %=4.07. SS: sum of squares, DF: degrees of freedom, MS: mean square.
